# Steroid-Responsive Chronic Schizophreniform Syndrome in the Context of Mildly Increased Antithyroid Peroxidase Antibodies

**DOI:** 10.3389/fpsyt.2017.00064

**Published:** 2017-04-21

**Authors:** Dominique Endres, Evgeniy Perlov, Anne Nicole Riering, Viktoria Maier, Oliver Stich, Rick Dersch, Nils Venhoff, Daniel Erny, Irina Mader, Ludger Tebartz van Elst

**Affiliations:** ^1^Faculty of Medicine, Department of Psychiatry, Section for Experimental Neuropsychiatry, University of Freiburg, Freiburg, Germany; ^2^Clinic for Psychiatry Luzern, Luzern, Switzerland; ^3^Faculty of Medicine, Department of Neurology, University of Freiburg, Freiburg, Germany; ^4^Faculty of Medicine, Department of Rheumatology and Clinical Immunology, University of Freiburg, Freiburg, Germany; ^5^Faculty of Medicine, Institute of Neuropathology, University of Freiburg, Freiburg, Germany; ^6^Berta-Ottenstein-Programme, Faculty of Medicine, University of Freiburg, Freiburg, Germany; ^7^Faculty of Medicine, Department of Neuroradiology, University of Freiburg, Freiburg, Germany

**Keywords:** Hashimoto encephalopathy, steroid-responsive encephalopathy associated with autoimmune thyroiditis, schizophrenia, thyroiditis, thyroid peroxidase, corticosteroids

## Abstract

**Background:**

Schizophreniform syndromes can be divided into primary forms from polygenic causes or secondary forms due to immunological, epileptiform, monogenic, or degenerative causes. Steroid-responsive encephalopathy associated with autoimmune thyroiditis (SREAT) is a secondary immunological form associated with increased thyroid antibodies, such as antithyroid peroxidase antibodies and shows a good response to corticosteroids.

**Case presentation:**

We present the case of a 41-year-old woman suffering from a schizophreniform syndrome. Starting at the age of 35, she developed psychotic exacerbations with formal thought disorder, acoustic hallucinations, cenesthopathic experiences, and loss of ego boundaries. At the same time, she began to suffer from chronic sexual delusions and olfactory hallucinations, which did not respond to neuroleptic medication. Her levels of antithyroid peroxidase antibodies were slightly increased, and the blood–brain barrier was disturbed. An electroencephalogram (EEG) showed intermittent generalized slowing, and cerebral magnetic resonance imaging (cMRI) depicted mild temporolateral atrophy. High-dose corticosteroid treatment led to convincing improvement of attentional performance and the disappearance of delusions and olfactory hallucinations.

**Conclusion:**

SREAT can mimic typical symptoms of schizophreniform syndromes. The increased titer of antithyroid peroxidase antibodies in combination with the EEG slowing, blood–brain barrier dysfunction, and the cMRI alterations were the basis for suspecting an immunological cause in our patient. Chronic delusions, olfactory hallucinations, and cognitive deficits were successfully treated with corticosteroids. The occurrence of secondary immunological forms of schizophreniform syndromes demonstrates the need for innovative immunosuppressive treatment options.

## Background

Schizophreniform syndromes are common severe disorders that are characterized by delusions, hallucinations, loss of ego boundaries, cognitive deficits, impaired motivation, and social withdrawal (1). Primary schizophrenia, which has polygenic causes, can be distinguished from various secondary forms of schizophreniform syndromes caused by immunological, epileptiform, monogenic, or degenerative factors. Immunological encephalopathies can be associated with antibodies against thyroid tissue, such as steroid-responsive encephalopathy associated with autoimmune thyroiditis (SREAT) with antithyroid peroxidase antibodies, intracellular onconeural or synaptic antigens, such as limbic encephalitis with anti-Hu antibodies, or neuronal cell surface antigens, such as autoimmune encephalitis due to anti-*N*-methyl-d-aspartate receptor (NMDAR) antibodies (2, 3). SREAT should be considered in the context of neuropsychiatric symptoms, autoimmune thyroiditis, increased thyroid antibodies, such as antithyroid peroxidase or anti-thyroglobulin antibodies, and other organic alterations [e.g., blood–brain barrier dysfunction in the cerebrospinal fluid, abnormal encephalopathic patterns as identified by an electroencephalogram (EEG), and non-specific white matter lesions as identified by cerebral magnetic resonance imaging (cMRI) (4–6)]. SREAT can mimic schizophreniform and other psychiatric syndromes (3, 7).

## Case Presentation

We present the case of a single 41-year-old female teacher and sports therapist suffering from a schizophreniform syndrome. In her third decade, she developed recurrent reactive depressive episodes. In her fourth decade, she developed a sexualized delusional system, which chronified. During this time, she suffered from repeated episodes with hallucinations.

The patient suffered reactive depressive episodes at 22, 28, and 32 years of age. At age 32, she experienced delusions for the first time. At age 35, she experienced her first schizophreniform episode with delusions, hallucinations, and promiscuity. During this episode, her mood was hypomanic. Since that time, she has suffered from a complex system of chronic delusions and olfactory hallucinations. At 40 and 41 years of age, she developed exacerbations with hallucinations. During these episodes, she suffered from formal thought disorder (long, incoherent train of thought) and auditory hallucinations. During the auditory hallucinations, she heard the voices of her neighbors ask her to have sexual intercourse and masturbate, the voice of a man who talked about the sexual lives of her friends, and a computer voice that told her which clothes to wear and that she was a “porn queen.” She also had cenesthopathic experiences in which she felt that she was being irradiated, her breasts were tense, and her intestines were pulsating, and felt that she was being externally controlled. In the latter case, she thought that she was being irradiated by the neighbors, her thoughts were being tracked, and she could feel the bodily sensations of her neighbors. These symptoms disappeared after she received neuroleptic treatment.

**Figure 1:**
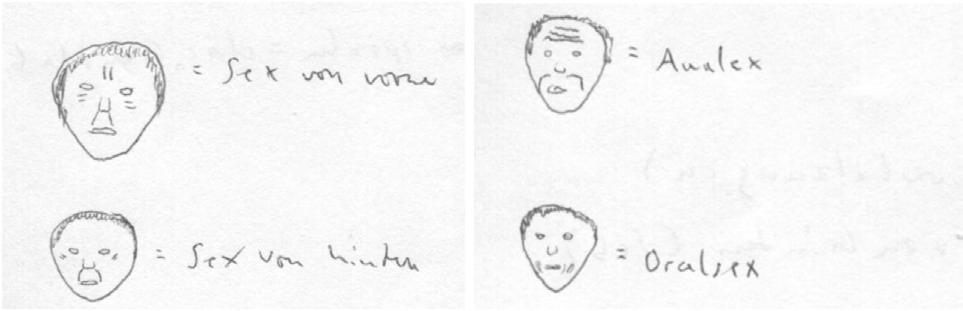
**The meaning of different wrinkles**. Top left: sexual intercourse from the front; top right: anal sex; lower left: sexual intercourse from behind; lower right: oral sex.

However, simultaneously at age 35, she developed a complex system of chronic delusions in which she attributed sexual body parts to everyday gestures. In this sexualized delusional system, the nostrils represented the buttocks, the mouth was the vagina, the tongue was the penis, and the ears were a combination of the bottom and the genitals. If a person scratched at their nose, our patient identified this gesture as proof that the person would like to have or that they had anal intercourse. The precise area of the nose that the person scratched was attributed to different positions of sexual intercourse. She had similar convictions if a person touched the mouth, tongue, or ears. Wrinkles at different locations in the face were assigned specific meanings with respect to sexual preferences (Figure 1). The patient was absolutely convinced of her interpretations. She also believed that she had the ability to smell whether people had had sexual intercourse and that other people could smell whether she had had sex. In addition, she had olfactory hallucinations in which she smelled urine, feces, and vaginal secretions in different situations. She took care not to wear provocative clothes to avoid attracting the sexual attention of men. All these symptoms persisted under treatment with neuroleptic medication.

### Developmental, Somatic, and Family History

The patient reported no history of *in utero* or birth complications, febrile convulsions, inflammatory brain diseases, or cerebral contusions. Her childhood development was normal, and there was no evidence of any neurodevelopmental disorder, such as attention-deficit hyperactivity disorder or autism. There was also no evidence of a personality disorder. Her somatic history was unremarkable, except that she had been diagnosed with Hashimoto’s thyroiditis. She also had no history of alcohol or drug abuse. There were no psychotic or neurological disorders in her family history, although her father did have a washing obsession, while her mother abused alcohol and her sister was diagnosed with recurrent depression.

### Investigations

**Figure 2:**
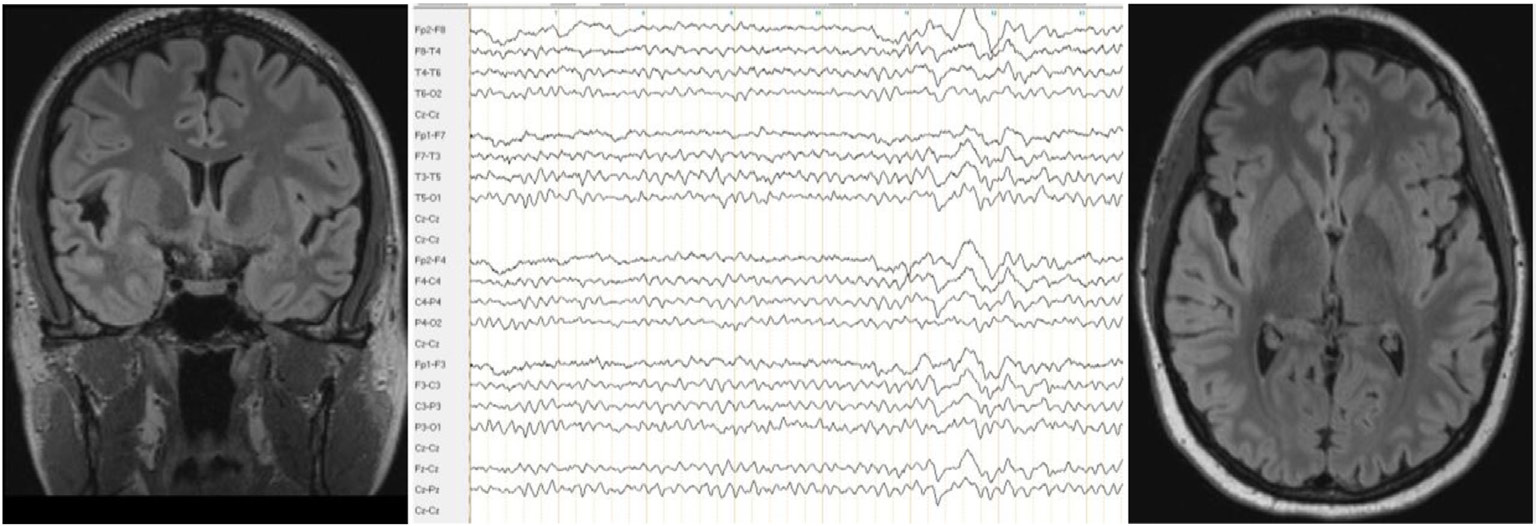
**The cerebral magnetic resonance imaging showed mild temporolateral atrophy, and the electroencephalogram showed intermittent generalized slow waves**.

**Figure 3:**
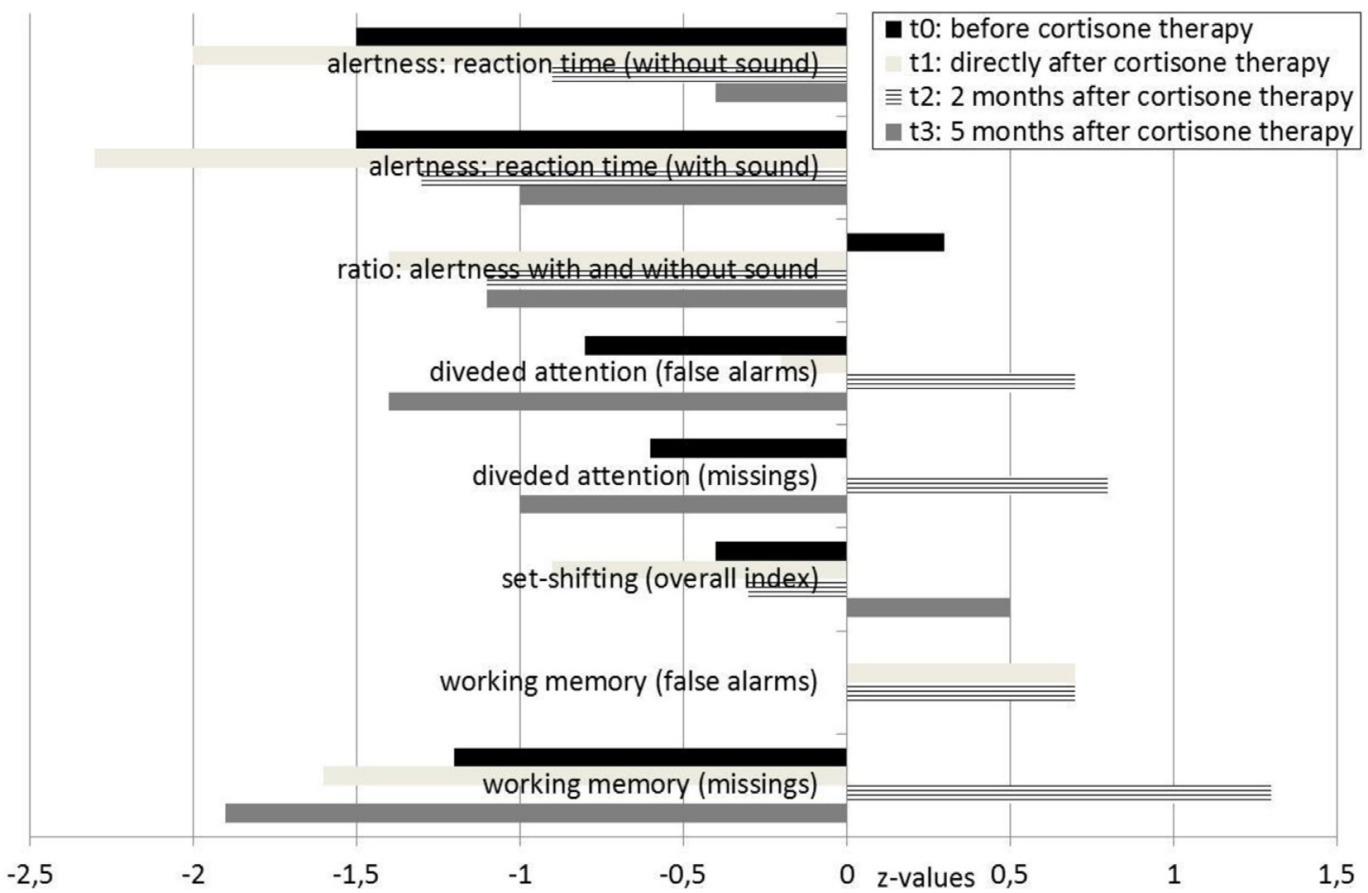
**Test for attentional performance**.

A neurological examination of the patient was normal. Serum analysis showed increased antithyroid peroxidase antibodies (35–55 IU/mL over the course of the disease and before steroid treatment; normal range <34 IU/mL). Levels of anti-thyroglobulin and antithyroid-stimulating hormone receptor antibodies were unremarkable. Thyroid-stimulating hormone levels were in the upper range; triiodothyronine and thyroxine levels were normal. Screening for rheumatoid factors, antinuclear antibodies, and antineutrophil cytoplasmic antibodies, as well as infectious diseases, such as *Borrelia*, lues, and HIV, showed no relevant abnormalities. In the cerebrospinal fluid analyses, we detected mild blood–brain barrier impairment and increased age-dependent albumin quotient (7.5; age-dependent reference <6.7 × 10^−3^). The white cell count (2/μL) and IgG index (0.43) were normal. No cerebrospinal fluid-specific oligoclonal bands were found. Antibodies against neuronal cell surface antigens [NMDAR, AMPA-R, GABA-B-R, and VGKC-complex (LGI1, Caspr2)] were negative in the cerebrospinal fluid. No antibodies against intracellular onconeural or synaptic antigens (Yo, Hu, CV2/CRMP5, Ri, Ma1/2, SOX1, GAD, and amphiphysin) were found in the serum. The cMRI showed a few right-accentuated hippocampus cysts in the cortical region. No white matter lesions were found. Mild temporolateral atrophy was identified. Repeated EEGs showed intermittent rhythmic delta activity (Figure 2). The neuropsychological test of attentional performance (TAP) revealed severe deficits in alertness and working memory (Figure 3).

### Differential Diagnosis

The symptoms met the ICD-10 criteria for paranoid hallucinatory schizophrenia (F20.0). Because the patient had Hashimoto’s thyroiditis and increased antithyroid peroxidase antibodies, we also considered SREAT.

### Treatment

Different neuroleptics (amisulpride, aripiprazole, clozapine, flupentixol, olanzapine, quetiapine, and risperidone) were used to successfully treat our patient’s acoustic and cenesthopathic experiences and loss of ego boundaries. However, average doses were not tolerated by the patient, and the neuroleptics were not able to treat the chronic delusional system and olfactory hallucinations. The same was true for additional treatment with lithium and valproic acid. The patient’s hypothyroidism was successfully treated with 100 µg thyroxine daily. Because of treatment resistance and organic abnormalities due to potential SREAT, we started treatment with 500 mg methylprednisolone administered intravenously once per day for five consecutive days. Methylprednisolone treatment was continued with 100 mg administered orally each day, which was then gradually tapered over nearly 3 months. Treatment with amisulpride (400 mg), lithium (450 mg), pipamperone (20 mg), thyroxine (100 µg), selenium (0.1 mg), and cholecalciferol (0.025 mg) was continued.

### Outcome and Follow-up

Following the high-dose methylprednisolone treatment, the patient experienced full remission of all psychotic symptoms, including the delusional system and olfactory hallucinations. After realizing that the former system was a delusion, our patient developed depression. She felt sad and embarrassed about her previous misinterpretations and the “wasted years” of the disease. TAP at that time showed deterioration (t1, Figure 3).

Approximately 3 weeks later, her mood normalized. A TAP was performed again 2 months after starting high-dose methylprednisolone treatment and showed that the patient’s alertness, divided attention, and working memory had all improved. However, the cMRI and EEG results were unchanged. Analysis of the cerebrospinal fluid showed that it normalized after steroid treatment; the blood–brain barrier dysfunction was no longer detected. Levels of antithyroid peroxidase antibodies were no longer elevated. Because of the positive response to the immunosuppressive treatment, we diagnosed SREAT encoded as an organic schizophreniform syndrome (ICD-10: F06.2). Six months later, the patient was still free of delusions, hallucinations, and loss of ego boundaries. Amisulpride treatment was reduced to 50 mg, pipamperone was stopped, and lithium, thyroxine, selenium, and cholecalciferol treatment remained unchanged. However, a TAP showed decreased results for divided attention and working memory (Figure 3). Antithyroid peroxidase antibody titers were still normal.

## Discussion

We present the case of a female patient with a chronic schizophreniform syndrome, who had side effects and insufficient responsiveness to neuroleptic medication, had mild organic alterations with increased levels of antithyroid peroxidase antibodies, and responded to treatment with corticosteroids. Therefore, we diagnosed the patient with SREAT, which is typically characterized by seizures (47%), confusion (46%), memory disturbances (43%), disordered speech (37%), gait disturbance (27%), delusions (25%), myoclonic jerks (22%), and depression (12%) (6). Clinical manifestations of isolated schizophreniform syndromes have been described for individual cases in the literature (8–12). Most case studies reported acute onset or repetitive episodes of SREAT (9–12).

The distinctive features of our case were chronic delusions and olfactory hallucinations, which were successfully treated with high doses of corticosteroids. The EEG slowing and blood–brain barrier dysfunction identified in our patient are frequent, but non-specific alterations that are found in over 80% of SREAT patients on a review level (6). We suggested in earlier studies that the EEG slowing might lead to clinical symptoms *via* local area network inhibition (3, 13). Blood–brain barrier dysfunction might allow potentially pathogenic autoantibodies to enter the central nervous system (CNS), thereby causing subtle CNS inflammation. A similar cause has been proposed for anti-NMDAR encephalitis (14). One earlier study showed cross reactivity between antithyroid peroxidase antibodies and cerebellar astrocytes (15). The role of the cerebellum in psychiatric symptoms has also been described (16, 17). Therefore, anti-thyroideal antibodies might have a direct pathophysiological role in the development of neuropsychiatric symptoms. However, Blanchin et al. (15) only showed antibody binding, but no neuronal damage. Therefore, the thyroid antibodies might alternatively function as an epiphenomenon, similar to the MRZ reaction in patients with multiple sclerosis (18), along with increased susceptibility to autoimmune conditions.

Most researchers favor the idea that thyroid antibodies do not play a relevant role in the development of neuropsychiatric symptoms (19, 20). Thyroid antibodies have been found in the serum of 13% of healthy individuals (21, 22). Isolated elevated levels of antithyroid peroxidase antibodies were found in 34% of SREAT cases (6). In our patient, autoantibody titers were only slightly increased; however, earlier studies have shown that antibody titers are not correlated with clinical severity (4–6). Our patient had no white matter lesions, which have been described in up to 52% of SREAT patients (6). However, our patient did have mild temporolateral atrophy; atrophy has been described in earlier SREAT cases (7). Following the current diagnostic recommendations, SREAT can only be diagnosed by exclusion (22). Therefore, the presence of other well-characterized neuronal antibodies was excluded in our patient. However, it is possible that our patient’s symptoms might also be due to new or unknown antineuronal antibodies. An unbiased search on rodent brain sections could be an additional tool in future cases.

We treated our patient in a probatory manner with high-dose corticosteroids. According to prior research, in cases of autoimmune encephalitis, first-line therapy should include corticosteroids, intravenous immunoglobulins, or plasmapheresis, whereas second-line therapy should include rituximab or cyclophosphamide (23). In our patient, the blood–brain barrier disturbance and antithyroid peroxidase antibody titer normalized following corticosteroid therapy. Cognitive testing showed substantial improvement within 2 months, and the psychotic symptoms disappeared. If the autoantibodies played a direct pathophysiological role, closure of the blood–brain barrier would have helped avoid direct autoantibody-mediated effects in the CNS. Furthermore, one could speculate about alternative mechanisms. For example, the corticosteroid treatment could have led to epigenetic effects that modulated the genome functionality of different neural, glial, and immunological cell populations.

The worsening of neurocognitive testing after 5 months raises a question regarding the need for repeated or long-term immunosuppressive treatment. Patients with SREAT that have an insufficient response to corticosteroids are mostly treated with a second course of corticosteroids alone or combined with other immunosuppressive agents, such as azathioprine, intravenous immunoglobulins, or even plasmapheresis (6). For long-term immunosuppressive maintenance therapy, low-dose corticosteroids, azathioprine, methotrexate, cyclosporine A, mycophenolate mofetil, cyclophosphamide, rituximab, and intravenous immunoglobulins are available (23). Presently, there are no clear recommendations regarding long-term treatment for patients with SREAT. Therefore, further research is necessary in this field. Independent from such considerations, we found that neuroleptic treatment was initially sufficient for treating our patient’s acoustic hallucinations, cenesthopathic experiences, and loss of ego boundaries. Therefore, an (initial) combination therapy with neuroleptics and corticosteroids seems a reasonable treatment for patients with SREAT.

## Conclusion

Single cases of chronic schizophreniform symptoms in the context of autoimmune thyroiditis and slightly increased antithyroid peroxidase antibodies can be successfully treated with corticosteroids. However, these effects were only shown for a short-term follow-up in our patient; a diagnosis of cenesthopathic schizophrenia in our patient cannot be fully ruled out. We suggest that clinical screening for autoimmune conditions should become a routine procedure among patients with schizophreniform syndromes. The decision to administer immunosuppressive therapy in the presence of organ-specific autoantibodies should take into account other organic alterations, such as EEG and cMRI findings, or blood–brain barrier dysfunction. Therefore, cerebrospinal fluid analyses should also have a greater importance in patients with schizophreniform syndromes (24).

## Ethics Statement

The patient has given her signed written informed consent for this case report, including the presented images, to be published.

## Author Contributions

DEndres and LTvE treated the patient and performed the data research. VM supported the data research. DEndres wrote the paper. IM performed the cMRI analyses. RD performed the EEG analyses. OS performed the CSF analyses. NV performed the rheumatological analyses. ANR performed the neuropsychological testing. EP and DErny critically reviewed the diagnostic results and contributed to the manuscript preparation. All authors were critically involved in the theoretical discussion and composition of the manuscript; read and approved the final version of the manuscript.

## Conflict of Interest Statement

DEndres, EP, ANR, VM, RD, and DErny: None. OS: consulting and lecture fees, grant and research support from Bayer Vital GmbH, Biogen Idec, Genzyme, Merck Serono, Novartis, Sanofi-Aventis, and Teva. NV: advisory boards, lectures, research or travel grants within the last 3 years: Janssen-Cilag, Roche, Novartis, AbbVie, GSK, Medac, and Pfizer. IM: lecture fees from UCB Pharma GmbH, Germany. LTvE: advisory boards, lectures, or travel grants within the last 3 years: Eli Lilly, Janssen-Cilag, Novartis, Shire, UCB, GSK, Servier, Janssen, and Cyberonics.
